# Antifungal Activity of Isoliquiritin and Its Inhibitory Effect against *Peronophythora litchi* Chen through a Membrane Damage Mechanism

**DOI:** 10.3390/molecules21020237

**Published:** 2016-02-19

**Authors:** Jianjun Luo, Zhibin Li, Jingjing Wang, Qunfang Weng, Shaohua Chen, Meiying Hu

**Affiliations:** Key Laboratory of Natural Pesticide and Chemical Biology, College of Agriculture, South China Agricultural University, Guangzhou 510642, China; luojianjun@scau.edu.cn (J.L.); chibun_li@163.com (Z.L.); wangjingjing968@126.com (J.W.); wengweng@scau.edu.cn (Q.W.); csh-happy@163.com (S.C.)

**Keywords:** isoliquiritin, *Peronophythora litchi* Chen, antifungal, SEM, mechanism

## Abstract

This study investigated the antifungal activity and potential antifungal mechanism(s) of isoliquiritin against *P. litchi* Chen, one of the main litchi pathogens. The antifungal activity of isoliquiritin against *P. litchi* Chen had been proven in a dose-dependent manner through *in vitro* (mycelial growth and sporangia germination) and *in vivo* (detached leaf) tests. Results revealed that isoliquiritin exhibited significant antifungal activity against the tested pathogens, especially, *P. litchi* Chen, with a minimum inhibitory concentration of 27.33 mg/L. The morphology of *P. litchi* Chen was apparently changed by isoliquiritin through cytoplasm leakage and distortion of mycelia. The cell membrane permeability of the *P. litchi* Chen increased with the increasing concentration of isoliquiritin, as evidenced by a rise in relative electric conductivity and a decrease in reducing sugar contents. These results indicated that the antifungal effects of isoliquiritin could be explained by a membrane lesion mechanism causing damage to the cell membrane integrity leading to the death of mycelial cells. Taken together, isoliquiritin may be used as a natural alternative to commercial fungicides or a lead compound to develop new fungicides for the control of litchi downy blight.

## 1. Introduction

The litchi (*Litchi chinensis* Sonn.) is an exotic fruit in Southeast Asia, especially in China, where about 1.5 million tons of litchi are produced annually [1]. Litchi has a delicious taste and lovely shape, being desired by many people [2]. In the last 30 years, other semitropical regions have started to plant this kind of fruit [3]. Litchi production is increasingly important in these countries because the popularity of litchi is increasing in the global market. The area cultivated for litchi exceeds 600,000 hectares in China, and new areas are still being planted around the Asia-Pacific region [4].

**Figure 1:**
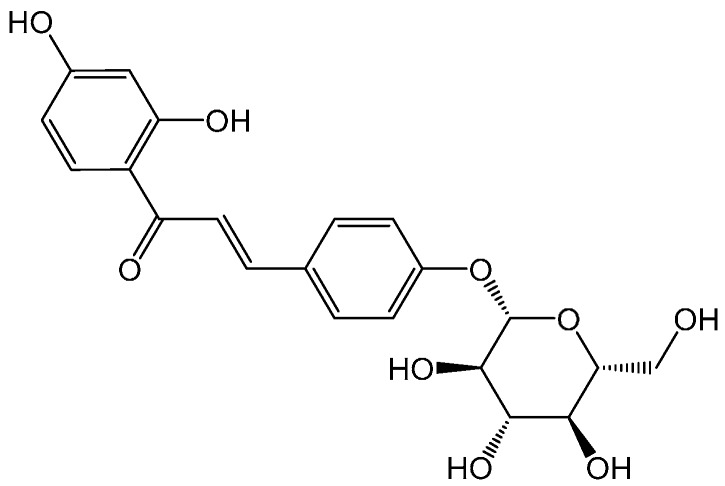
Structure of isoliquiritin.

Litchi downy blight, caused by *P. litchii* Chen, is one of the most important diseases of litchi, which not only damages ready-to-mature fruit, but also the inflorescences, twigs, young fruit, leaves, and root cortical tissues. The infection site produces withering and watery brown spots, then produces white aerial mycelium resulting in significant postharvest losses. Reduction of litchi production is mainly due to the spread of this disease during extensive hot, humid, and rainy weather, which often happens during the litchi fruit growth period in southern China [5,6,7]. Currently, the main therapy and prevention mean against *P. litchii* Chen is the use of chemicals. In the 1990s, multisite inhibitors (such as chlorothalonil) and the target site-specific (such as metalaxyl) were used to control this disease [8]. Mandipropamid [9], belonging to a novel fungicide group, the carboxyl acid amide (CAA) fungicides, was first registered in 1996 in China for the control of litchi downy blight [10,11]. Dimethomorph (DMM) and benthiavalicarb (valinamid carbamates), were effective against oomycete foliar plant pathogens, such as *P. litchii* Chen [12,13]. However, considering the increasing development of resistance by pathogens, carcinogenic risk, environmental pollution, and public concerns over food safety, some alternative means to control the litchi downy blight are urgently required. There are numerous plants which can produce secondary metabolites with insecticidal, antifungal or antibacterial biological activity, therefore, natural products can be used as ideal agents to control crop diseases. Liquorice is the dried roots and rhizomes that is isolated from *Glycyrrhiza* species (Leguminosae family). In China, only three species, *Glycyrrhiza uralensis* Fisch., *Glycyrrhiza glabra* L. and *Glycyrrhiza inflate* Bat., are officially used as traditional Chinese medicine (TCM) for their functions of relieving coughing, supplement the vital energy, tonifying spleen and stomach, alleviating pain, and eliminating phlegm [14]. In addition, it is also used in many other fields as a health food, flavoring agent, commodity, and tobacco additive [15]. The chemical constituents of liquorice play an important role in its bioactivities, and more than 400 compounds have been identified in *Glycyrrhiza* species. Plant-derived antimicrobial agents represent a group of medicinally important secondary metabolites, among which polyphenols are distributed in most higher plants [16,17]. Four major classes of polyphenols are found, namely phenolic acids, flavonoids, lignans and stilbenes [18]. Isoliquiritin (Figure 1) is one of flavonoid compounds of liquorice responsible for its bioactivity, and it displays a variety of pharmacological effects, such as antiangiogenic [19], antidepressant [20], p53-dependent pathway inhibition and crosstalk between kinase Akt activities [21], and it suppresses lipopolysaccharide (LPS)-induced inflammatory responses [22]. However, there is currently limited information on the antifungal activity of isoliquiritin.

In this study, we investigated the antifungal activity of isoliquiritin against *P. litchii* Chen *in vitro* and *in vivo*. The antifungal mechanism of isoliquiritin against *P. litchi* was also explored.

## 2. Results

### 2.1. Antifungal Activity

The *in vitro* effect of isoliquiritin on mycelial growth is shown in Table 1 and the toxicity measurement results are shown in Table 2. The mycelial growth of four plant pathogenic fungi was inhibited by isoliquiritin in a dose-dependent manner. Isoliquiritin had the best inhibition effect on *Phytophthora*, inhibiting half of the mycelial growth of *P. litchii* Chen (EC_50_ was 27.33 mg/L) and *P. capsici* Leonian (EC_50_ was 22.52 mg/L) even at a relatively low concentration (less than 28 mg/L), while inhibition of *S. sclerotiorum* (Lib.) de Bary and *C. herbarum* (Pers) LK. ex Fr. needed relatively higher concentrations (more than 291 mg/L). Isoliquiritin at 200 mg/L inhibited mycelial growth of *P. litchii* Chen and *P. capsici* Leonian, with radial growth inhibition values of 96.45% and 93.35%, while isoliquiritin showed probably half the inhibition of mycelial growth in *S. sclerotiorum* (Lib.) de Bary and *C. herbarum* (Pers) LK. ex Fr. Isoliquiritin at 400 mg/L completely inhibited of mycelial growth of *P. litchii* Chen.

### 2.2. Assay of Sporangia Germination and Influence of Sporangia Morphology

**Figure 2:**
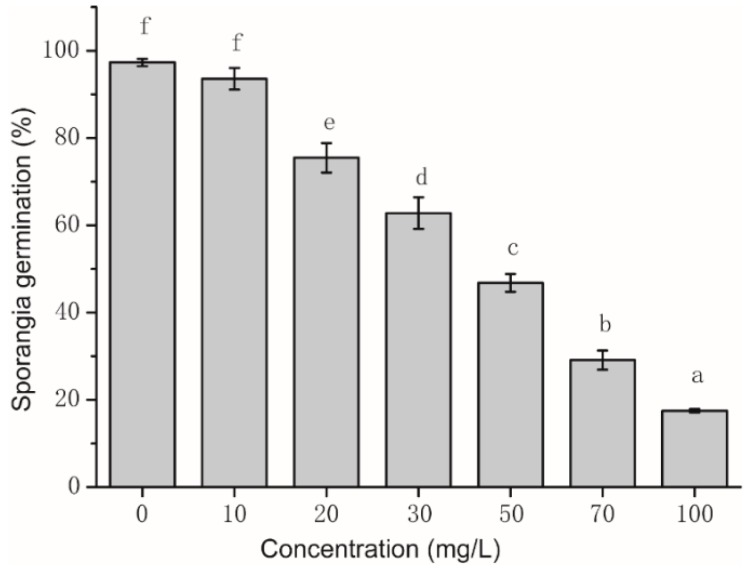
Inhibition effect of isoliquiritin against *P. litchii* Chen sporangia germination. Values were presented as mean ± S.E. Data presented were the means of pooled data (*n* = 6). The column with different lowercase letters between different concentrations indicates significant differences according to Duncan Multiple Range Test (*p* < 0.05).

The inhibitory effect of isoliquiritin (Figure 2) on sporangia germination of *P. litchii* Chen was positively related to the concentration of isoliquiritin used. Also, all *P. litchii* Chen sporangia germinated after 4 h of incubation at 25 °C ± 1 °C in PDA without isoliquiritin. Sporangia germination was significantly inhibited by isoliquiritin when the concentration was 20 mg/L or more. When the concentration of isoliquiritin reached 70 mg/L and 100 mg/L, the inhibitory effects of isoliquiritin on sporangia germination were 70.89% and 82.5%, respectively (data not shown).

**Figure 3:**
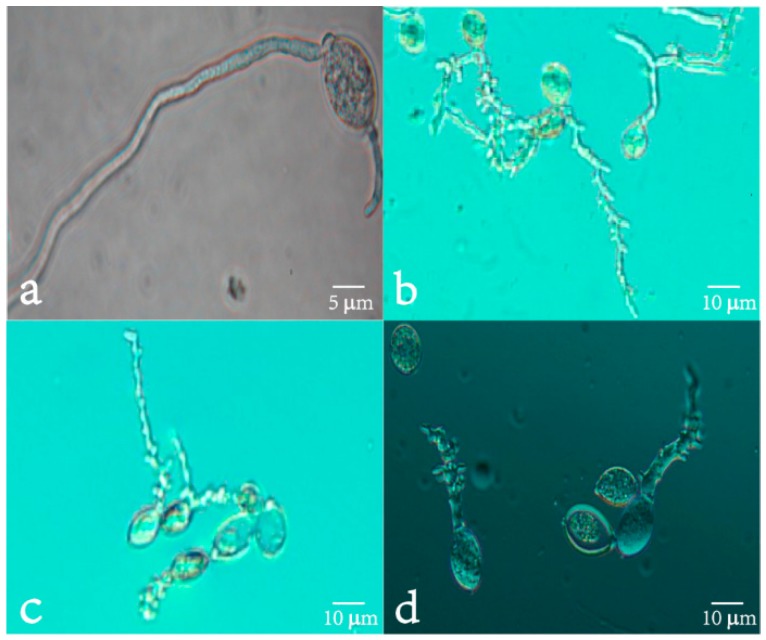
Micrographs of the sporangia germination of *P. litchii* Chen with or without isoliquiritin. (**a**) Control; (**b**) 30 mg/L; (**c**) 50 mg/L; and (**d**) 70 mg/L.

The effect of isoliquiritin on the morphology of sporangia germination of *P. litchii* Chen was examined using optical microscopy (Figure 3). The control sporangia grown on sterile water had smooth, slender, little branch germ tube. In contrast, all sporangia of *P. litchii* Chen treated with 30, 50, 70 μg/mL of isoliquiritin for 4 h showed considerable changes in sporangia morphology. *P. litchii* Chen treated with 30, 50, 70 μg/mL of isoliquiritin showed deformed germ tubes. Although some of sporangia germinated, all of the germ tubes had a morphology of chunky, nodular swelling and distortion, multi-site germination and germ tube base multi-branch.

### 2.3. Detached Leaf Test

**Figure 4:**
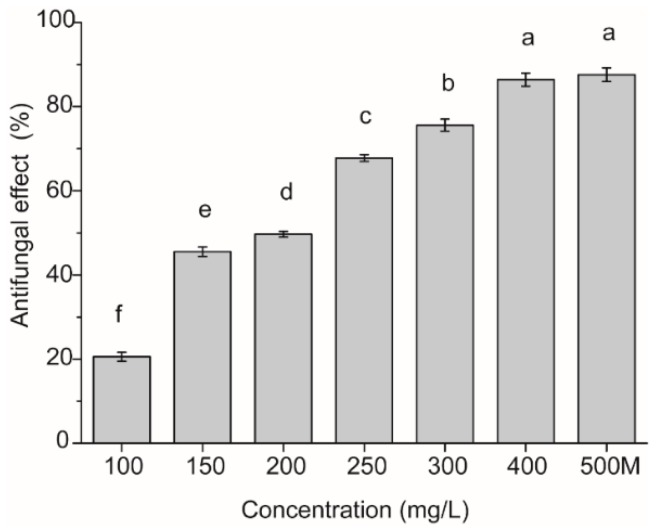
Inhibition effect of isoliquiritin against *P. litchii* Chen by detached leaf test. 500M: 25% metalaxyl-propamocarb WP diluted 500 times. Values were presented as mean ± S.E. Data presented were the means of pooled data (*n* = 6). The column with different lowercase letters between different concentrations indicates significant differences according to Duncan Multiple Range Test (*p* < 0.05).

The effect of isoliquiritin against *P. litchii* Chen by the detached leaf test is presented in Figure 4. The inhibitory effect of isoliquiritin against *P. litchii* Chen was positively related to the concentration of isoliquiritin. All detached leaves showed disease symptoms after 72 h of incubation at 25 °C ± 1 °C without isoliquiritin. Symptoms on detached leaves were significantly inhibited by isoliquiritin when the concentration was 100 mg/L or more. When the concentration of isoliquiritin reached 300 mg/L, the inhibitory effect reached more than 75%. Furthermore, the inhibitory effect of isoliquiritin at 400 mg/L was 86.42%, which corresponded to the effect of 25% metalaxyl-propamocarb WP diluted 500 times.

### 2.4. Scanning Electron Microscopy (SEM)

**Figure 5:**
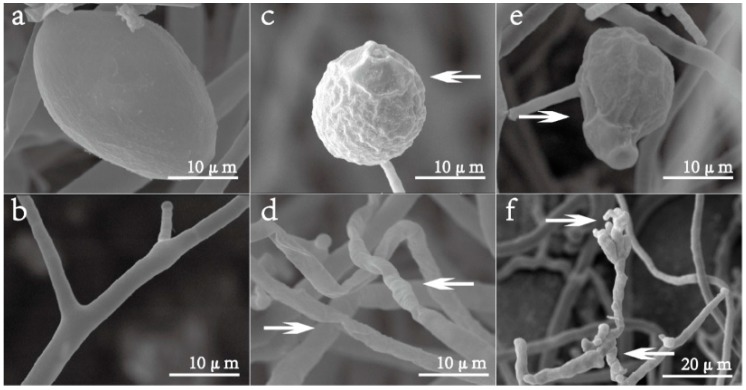
Effect of isoliquiritin on mycelium morphological changes of *P. litchii* Chen. (**a**,**b**): *P. litchii* Chen without isoliquiritin (control); (**c**,**d**): *P. litchii* Chen with isoliquiritin (40 mg/L); (**e**,**f**): *P. litchii* Chen with isoliquiritin (80 mg/L).

The effect of isoliquiritin on the morphology of *P. litchii* Chen was examined using SEM (Figure 5). The sporangia of *P. litchii* Chen grown on PDA had normal, oval, plump and homogenous morphology (Figure 5a), and the hyphae of control fungus growing on PDA were also normal, tubular, regular, and homogeneous (Figure 5b). All mycelia and sporangia of *P. litchii* Chen treated with 40 mg/L or 80 mg/L of isoliquiritin for 7 d showed considerable changes in their morphology. *P. litchii* Chen treated with 40 mg/L of isoliquiritin showed a warty surface of sporangia (Figure 5c) and a collapse and shrunken of mycelia (Figure 5d). In contrast, the sporangia of *P. litchii* Chen treated with 80 mg/L of isoliquiritin appeared severely collapsed because of the lack of cytoplasm (Figure 5e). Moreover, the mycelia showed a morphology of swelling and malformation (Figure 5f). Shrunken and distorted mycelia were also observed (Figure 5f).

### 2.5. Measurement of Relative Electric Conductivity

**Figure 6:**
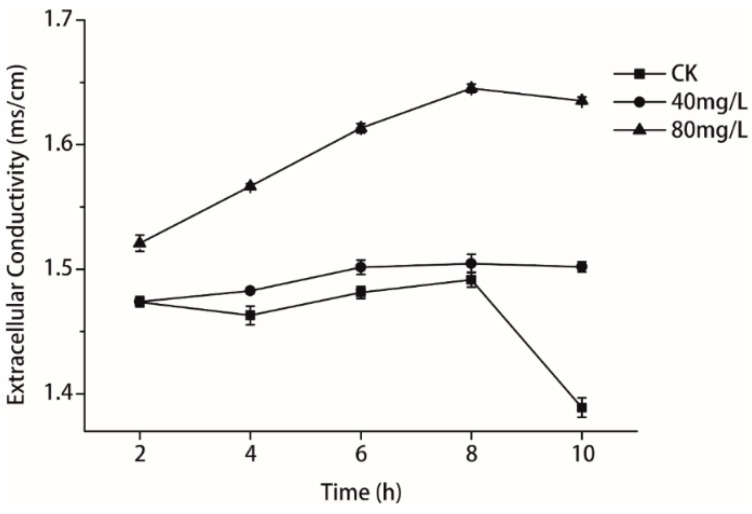
Effects of isoliquiritin on the relative electric conductivity of *P. litchii* Chen. Values were mean (*n* = 3) ± S.E.

Intracellular soluble matter leaked from *P. litchii* Chen cells incubated with isoliquiritin (Figure 6). In general, 80 mg/L of isoliquiritin progressively induced the release of intracellular soluble matter during the experimental period of treatment, and the conductivity reached its peak, which was 1.645 ms/cm, after 8 h. When the incubation time increased to 10 h, 80 mg/L of isoliquiritin had no further effect. By contrast, intracellular soluble matter of fungal cells by incubation with isoliquiritin at 40 mg/L was slightly more than from the control between 2 h and 8 h. After 8 h of incubation, conductivity by 40 mg/L of isoliquiritin reduced modestly and reached 1.502 ms/cm after 10 h of incubation.

### 2.6. Determination of Reducing Sugar

**Figure 7:**
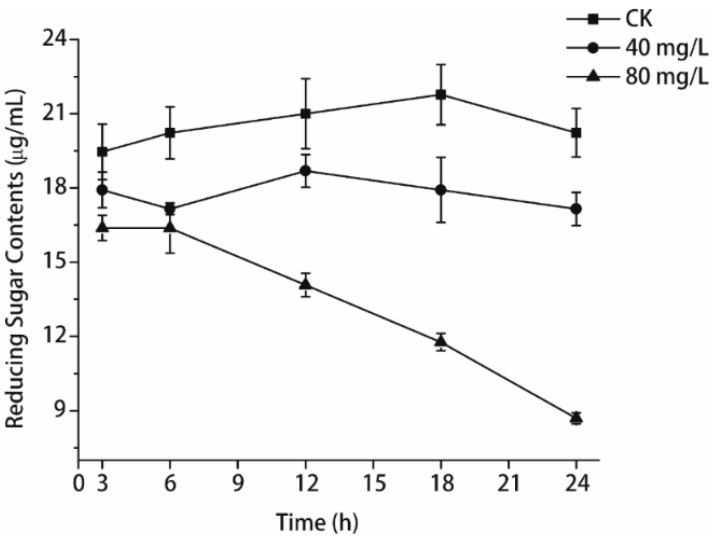
Effects of isoliquiritin on the reducing sugar contents of *P. litchii* Chen. Values were mean (*n* = 3) ± S.E.

The reducing sugar contents in *P. litchii* Chen with isoliquiritin continuously decreased during the entire period, whereas those in the untreated cells remained stable (Figure 7). The reducing sugar contents of *P. litchii* Chen incubated with 40 mg/L and 80 mg/L of isoliquiritin for 3 h were 17.92 and 16.38 μg/mL, respectively, which were significantly lower than that of the control (19.46 μg/mL). During the experiment, the reducing sugar contents of *P. litchii* Chen incubated with isoliquiritin were lower than the control, and this change became more evident with increasing exposure time. At 24 h of exposure, the reducing sugar contents in *P. litchii* Chen treated with 40 mg/L and 80 mg/L of isoliquiritin were 17.15 and 8.69 μg/mL, respectively, which were significantly lower than that of the control (20.23 μg/mL).

## 3. Discussion

It was known that liquorice, because of its health care and antisepsis effects, had been applied to China as a flavoring agent in order to impart extra flavor to food. Plant-based secondary metabolites such as essential oils, odorous and volatile products, have been widely used in the food industry and medical research [23].

Polyphenols can play a role in different fields, functioning as antioxidant, antimicrobial, anti-allergic, anti-inflammatory and anticancer agents [24]. For example, Jatinder *et al.* [17] reported that the polyphenol extract of jambolan exhibited a broad spectrum antimicrobial activity against Gram-positive bacteria (*Staphylococcus* aureus and MRSA), Gram-negative bacteria (*Escherichia coli* and *Klebsiella* pneumoniae) and *Candida albicans* using the agar well diffusion method. Renuka, Umesh, Kiran, and Satish [25] found that the EC_50_ value of polyphenolics from the roots of *Bauhinia racemosa* for growth inhibition of *Aspergillus flavus*, was 1.95 mg/L. In our study (Table 1 and Table 2) isoliquiritin exhibited a broad spectrum antifungal activity against phytopathogenic fungi.

Both the mycelial growth and the sporangia germination of *P. litchii* Chen were inhibited in the presence of isoliquiritin, and the inhibitory efficacy was positively correlated with the isoliquiritin concentration. The results (Table 1) showed that the mycelial growth of *P. litchii* Chen was totally suppressed, when the concentration of isoliquiritin was 400 mg/L, and the sporangia germination of *P. litchii* Chen (Figure 2) was significantly inhibited by isoliquiritin at 100 mg/L. It (Figure 3) also was observed that the morphology of germ tubes became abnormal upon exposure to isoliquiritin. The finger millet (*Eleusine coracana*) seed coat extract tested in previous researches [26] showed higher antifungal activity against *Aspergillus flavus*, and their results showed that finger millet seed coat can probably be utilised as an alternative natural antioxidant and food preservative. Similarly, Shangmugen, and Thangaraj [27], who found that acetone extract of polyphenols by *Passiflora ligularis* inhibited *Aspergillus niger* (13.91 mm) in the disc diffusion method. The above studies point out that plant extract polyphenols, including isoliquiritin of course, have significant antifungal activity.

The results of *in vivo* investigations showed that isoliquiritin concentrations of 100–400 mg/L had an obvious inhibitory effect against *P. litchii* Chen in detached leaves. A positive effect was exhibited by isoliquiritin on leaves of litchi, and it could reduce the severity of lesions, with maximum effectiveness when the concentration was 400 mg/L or more. The inhibitory effect against *P. litchii* Chen in detached leaves by isoliquiritin at 400 mg/L was similar to that obtained using 25% metalaxyl- propamocarb WP diluted 500 times. There are only few reports that similarly focus on isoliquiritin for inhibiting phytopathogenic fungi. The SEM images (Figure 5) clearly showed the difference between the treated samples and control mycelia and sporangium of *P. litchii* Chen, showing a warty sporangia surface and shrunken mycelia after exposure to 50 mg/L of isoliquiritin, and the phenomenon became more serious when isoliquiritin was used at a relatively higher concentration (80 mg/L). These findings were in agreement with previous results reported by Jatinder *et al.* [17]. The changes of mycelia may be attributed to an increase in cell permeabilization. Thus, the fundamental mechanism the antifungal action of isoliquiritin against *P. litchii* Chen may be through membrane disruption and cell growth hindrance.

The relative electric conductivity test was applied in our study to illustrate the mechanism of antifungal action regarding the membrane permeability of *P. litchii* Chen. Maintenance of ion homeostasis plays an important role in maintaining the energy status of the cell as it is significant to energy-relevant processes, such as solute transport, control of metabolism, management of turgor pressure and motility [28]. Hence, even relatively minor changes to the structure of cell membranes can generate harmful effects on cell metabolism and lead to cell death [29]. In this study, the relative electric conductivity of *P. litchii* Chen when exposed to isoliquiritin was measured at 40 mg/L and 80 mg/L levels. The results (Figure 6) evidently showed that the relative electric conductivity increased with increasing concentration of isoliquiritin, which distinctly meant that the membrane permeability injuries caused by isoliquiritin led to the leakage of ions. According to the experiment, we could conclude that the membrane structure of *P. litchii* Chen was severely damaged by isoliquiritin.

The reducing sugar content of *P. litchii* Chen was measured when the concentration of isoliquiritin was 40 mg/L and 80 mg/L, respectively. The results (Figure 7) demonstrated that the reducing sugar contents decreased with increasing concentration of isoliquiritin. This study indicated isoliquiritin was a stressor for *P. litchii* Chen which could bring about sugar starvation conditions and induce stress-activated protein release, interrupt the metabolism and transport of substances and finally lead to mycelium growth inhibition [30,31,32].

## 4. Experimental Section

### 4.1. Fungal Species

The fungal pathogen *P. litchi* Chen, *Phytophthora capsici* Leonian, *Sclerotinia. sclerotiorum* (Lib.) de Bary and *Cladosporium herbarum* (Pers) LK. ex Fr. were provided by the Department of Plant Pathology, College of Natural Resources and Environment, South China Agricultural University (Guangzhou, China) and maintained on potato dextrose agar (PDA) at 25 °C ± 1 °C. The concentration of sporangia of *P. litchi* Chen that was incubated at 25 °C ± 1 °C for 5 days was adjusted to 5 × 10^5^ cfu/mL use a haemocytometer.

### 4.2. Plant Material

The tender leaves of *Litchi chinensis* Sonn were collected from the local area of Guangzhou, Guangdong Province, China in March 2005. All the leaves were gathered from young shoots of *Litchi chinensis* Sonn and were of similar stage, shape and size. Leaves were cleaned with distilled water and dried at room temperature before testing.

### 4.3. Chemicals

Isoliquiritin (purity ≥ 98%) was purchased from Chengdu Must Bio-Technology Co., Ltd (Sichuan, China). 25% metalaxyl-propamocarb WP was purchased from Zhejiang Heben Pesticide Chemical Co., Ltd. (Zhejiang, China); 3,5-dinitrosalicylic acid (DNS) was purchased from Shanghai Chemical Reagent Company of the China Pharmaceutical Group (Shanghai, China). All reagents used in the study were of analytical grade.

### 4.4. Antifungal Activity

The effects of isoliquiritin on mycelial growth of *P. litchi* Chen were tested *in vitro* by the agar dilution method [33]. PDA (50 mL) was poured into sterilized Petri dishes (90 mm diameter) and measured amounts of isoliquiritin (with 75% methanol) were added to the PDA media to give the desired concentrations of 0, 1, 5, 10, 50, 100, 200, and 400 mg/L. A 6 mm diameter disc of inocula was cut from the periphery of an actively growing culture on PDA plates with a punch, and then was placed at the center of each fresh Petri plate. Culture plates were then incubated at 25 °C ± 1 °C for 5 days. Each treatment was performed in triplicates. The percentage of inhibition of mycelial growth (IMG) was calculated according to the following formula:

$$\text{IMG}\left(\%\right)=\frac{\text{dc}-\text{dt}}{\text{dc}}\times 100$$

where dc (cm) was the mean colony diameter for the control sets and dt (cm) was the mean colony diameter for the treatment sets.

### 4.5. Assay of Sporangia Germination and Influence of Sporangia Morphology

Sporangia used for these experiments were collected from 5-day-old cultures of fungi growing in PDA medium. The sporangia were collected from the plates with 1 mL sterile distilled water containing 0.1% Tween-80, filtered through four layers of sterile cheesecloth to remove hyphae, counted by a hemocytometer, and immediately used in the next test. An aliquot (10 μL) of *P. litchi* Chen sporangia suspension (5 × 10^5^ cfu/mL) was incubated into 10 μL PDA medium in separate depression slides. Then, the 10 μL amounts of isoliquiritin (with 75% methanol) dissolved in 0.1% Tween-80 was respectively transferred into the above culture medium to obtain 0, 10, 20, 30, 50, 70 and 100 mg/L concentrations. The depression slides were then put into plates with filter paper moistened with sterilized water on the bottom, and were incubated at 25 °C ± 1 °C for 4 h. A sporangium was considered germinated when the germ tube was equal to or greater than the diameter of the sporangium. About 200 sporangia were examined microscopically (BX51; Olympus, Tokyo, Japan). Germinated sporangia were expressed as a percentage of the total number of evaluated sporangia. After continuing incubation at 25 °C ± 1 °C for 4 h, sporangia morphology were observed under the microscope and recorded. Each treatment was performed in triplicates.

### 4.6. Detached Leaf Test

Litchi leaves of similar shape, size and age were used for these experiments, then washed by distilled water and dried at room temperature. Isoliquiritin (with 75% methanol) was dissolved in distilled water that had 0.1% Tween-80 to obtain 0, 100, 150, 200, 250, 300, 350, 400 mg/L concentrations. 25% Metalaxyl-propamocarb WP diluted 500 times was applied as positive control. In each treatment, 10 leaves were immersed into the drug-containing solution for 10 min and dried at room temperature. The treated leaves (back-to-front) were placed on the wet filter paper of Petri dish (150 mm diameter). The sporangia were collected from the plates with sterile distilled water, filtered through four layers of sterile cheesecloth to remove hyphae, counted by a hemocytometer, and immediately used in the next test. Sporangia suspension (5 × 10^5^ cfu/mL) was sprayed on the back of leaves with a hand-operated sprayer, and the leaves were incubated at 25 °C ± 1 °C for 72 h. Each assay contained three replicates for each concentration. Treatments were scored using disease index level (DIL) as follows: Level 0, the percentage of infection area of the entire leaf is 0; Level 1, less than 5%; Level 3, more than or equal to 5%, less than 10%; Level 5, more than or equal to 10%, less than 20%; Level 7, more than or equal to 20%, less than 50%; Level 9, more than or equal to 50%. According to Equations (1) and (2) calculate the incidence of the disease index (DI) and antifungal effect (AE).

(1) $$\text{DI}=\frac{\sum \text{DIL}\times \text{N}}{9\times \text{sum}}\times 100$$

(2) $$\text{AE}\left(\%\right)=\frac{{\text{DI}}_{0}-{\text{DI}}_{t}}{{\text{DI}}_{0}}\times 100$$

where N was the mean the number of leaves of disease index level sets, *sum* was the mean the total number of leaves sets, DI_0_ was the mean disease index of control sets and DI_t_ was the mean disease index of treatment sets.

### 4.7. Scanning Electron Microscopy (SEM)

The 5-day-old fungal cultures on PDA medium treated with isoliquiritin at 40 mg/L and 80 mg/L were used for all SEM observations [33,34]. About 5 mm × 5 mm segments were cut from cultures growing on PDA medium and promptly placed in vials containing 2.0% (*v*/*v*) glutaraldehyde in 0.05 mol/L phosphate buffer saline (pH 6.8) at 4 °C. Samples were kept in this solution for 48 h for fixation and then washed with distilled water three times for 20 min each. Following which they were dehydrated in an ethanol series (30%, 50%, 70%, and 95%, *v*/*v*), for 20 min in each alcohol dilution and finally with absolute ethanol for 45 min. Samples were then critical point dried in liquid carbon dioxide. Fungal segments were placed in desiccators until further use. Following drying, the specimen was sputter-coated with gold in an ion coater for 2 min, followed by microscopic examinations (XL-30 SEM; Philips-FEI, Amsterdam, The Netherlands).

### 4.8. Measurement of Relative Electric Conductivity

The permeability of *P. litchi* Chen cell membranes was expressed in terms of their electric conductivity, and was determined following the method described by Paul *et al.* [35]. Briefly, an aliquot (100 μL) of *P. litchi* Chen sporangium suspension (5 × 10^5^ cfu/mL) was inoculated in 20 mL of PDB medium. After incubation for 7 d at 25 °C ± 1 °C, 110 rpm, the fungal cells were centrifuged at 4000 *g* for 10 min and washed twice with sterilized water; the hyphae pellet was then resuspended in 20 mL of sterilized water. Isoliquiritin at different concentrations (40 and 80 mg/L) was added to the above resuspended solution and the electric conductivity of the mixtures was determined using a conductivity meter (DDS-11A; Shanghai Leici Instrument Inc., Shanghai, China) at 2, 4, 6, 8 and 10 h. In the control experiment, no isoliquiritin treatment was applied. Each assay contained three replicates for each concentration. Results were expressed as the amount of relative electric conductivity (ms/cm).

### 4.9. Determination of Reducing Sugar

Reducing sugar content was analyzed by the 3,5-dinitrosalicylic (DNS) colorimetric method [36,37] as described in Section 4.8. Isoliquiritin at different concentrations (40 and 80 mg/L) was added to the above resuspended solution. The solutions were incubated at 25 °C ± 1 °C, 110 rpm, for 3, 6, 12, 18 and 24 h. Then they were centrifuged at 4000 *g* for 10 min to obtain supernatants. No isoliquiritin treatment was applied in the control. For each of the 2 mL of the supernatants, 1.5 mL of DNS reagent was added. The mixture was heated in boiling water for 5 min until the red brown color was developed and cooled to room temperature in a water bath. The absorbance of the mixture was measured at 520 nm and the concentration of reducing sugars was calculated based on a standard curve obtained with D-glucose. Each assay contained three replicates for each concentration.

### 4.10. Statistical Analysis

A completely randomized design was used for all treatments. Data were statistically analyzed using analysis of variance (ANOVA). Data were presented as mean ± standard error (SE). The mean separations were carried out using Duncan’s multiple range tests and significance was determined at 0.5% level (SPSS 18.0, SSPS Inc., Chicago, IL, USA) [38].

## 5. Conclusions

In this research, it demonstrated that isoliquiritin, a metabolite of liquorice, could significantly inhibit the mycelial growth and spore germination of *P. litchii* Chen and reduce the disease on detached leaves. Isoliquiritin could inhibit the growth of *P. litchii* Chen by severely disrupting the membrane integrity of the fungal pathogen, leading to the leakage of intracellular contents. This suggests that isoliquiritin is a promising plant-derived bio-fungicide, and could be used as a lead compound to develop new fungicides.

## Figures and Tables

**Table 1:** Effect of isoliquiritin on four plant pathogenic fungi mycelial growth.

Concentration (mg/L)	Inhibition Activity (%)			
	PL	PC	SS	CH
1	3.98 ± 0.74 d	9.09 ± 0.39 e	20.84 ± 0.69 bc	20.39 ± 0.15 b
5	10.86 ± 0.39 cd	18.17 ± 1.08 d	24.17 ± 0.35 bc	22.84 ± 0.36 b
10	22.61 ± 0.89 c	33.71 ± 0.41 c	27.93 ± 0.61 bc	26.59 ± 0.86 b
50	66.29 ± 0.87 b	63.85 ± 0.41 b	41.46 ± 0.34 b	34.80 ± 0.76 b
100	83.58 ± 0.31 ab	81.82 ± 0.37 ab	48.12 ± 0.47 b	40.13 ± 0.35 ab
200	96.45 ± 0.20 a	93.35 ± 0.04 a	51.21 ± 0.39 ab	43.45 ± 0.33 ab
400	100.00 a	97.34 ± 0.39 a	64.31 ± 1.01 a	53.22 ± 0.41 a

PL: *P. litchii* Chen; PC: *P. capsici* Leonian; SS: *S. sclerotiorum* (Lib.) de Bary; CH: *C. herbarum* (Pers) LK. ex Fr. Values were presented as mean ± S.E. Data presented were the means of pooled data (*n* = 3). The column with different lowercase letters between different concentrations indicates significant differences according to Duncan Multiple Range Test (*p* < 0.05).

**Table 2:** Effect of isoliquiritin on four plant pathogenic fungi toxicity measurement results.

Fungal Pathogen	Y = a + bx	Correlation Coefficient	EC_50_ (mg/L)	95% Confidence Limits (mg/L)
PL	Y = 1.7583 + 2.2563x	0.9791	27.33	15.49–48.23
PC	Y = 2.9663 + 1.5035x	0.9924	22.52	19.37–44.17
SS	Y = 3.0569 + 0.7884x	0.9907	291.42	176.08–356.28
CH	Y = 3.9245 + 0.4195x	0.9836	367.79	268.24–417.65

PL: *P. litchii* Chen; PC: *P. capsici* Leonian; SS: *S. sclerotiorum* (Lib.) de Bary; CH: *C. herbarum* (Pers) LK. ex Fr. Data presented were the means of pooled data (*n* = 3).
